# Predicting Gram-Positive Bacterial Protein Subcellular Location by Using Combined Features

**DOI:** 10.1155/2020/9701734

**Published:** 2020-08-02

**Authors:** Feng-Min Li, Xiao-Wei Gao

**Affiliations:** College of Science, Inner Mongolia Agricultural University, Hohhot 010018, China

## Abstract

There are a lot of bacteria in the environment, and Gram-positive bacteria are the most common ones. Some Gram-positive bacteria are very harmful to the human body, so it is significant to predict Gram-positive bacterial protein subcellular location. And identification of Gram-positive bacterial protein subcellular location is important for developing effective drugs. In this paper, a new Gram-positive bacterial protein subcellular location dataset was established. The amino acid composition, the gene ontology annotation information, the hydropathy dipeptide composition information, the amino acid dipeptide composition information, and the autocovariance average chemical shift information were selected as characteristic parameters, then these parameters were combined. The locations of Gram-positive bacterial proteins were predicted by the Support Vector Machine (SVM) algorithm, and the overall accuracy (OA) reached 86.1% under the Jackknife test. The overall accuracy (OA) in our predictive model was higher than those in existing methods. This improved method may be helpful for protein function prediction.

## 1. Introduction

The cell is the most basic unit of life, and it contains many protein molecules. When a protein is in the right subcellular position, it can perform the right function [1]. So, studying protein subcellular location can help us better understand the biological function of proteins at the cellular level. In the postgenetic era, the amount of biological information has grown rapidly and the traditional experimental method became time-consuming and exhausting. So, the prediction of protein subcellular location based on the machine method has gradually become a hot research topic in bioinformatics [2–7].

Gram-positive bacteria are those that retain their original blue-violet color after being stained by Gram staining. Gram-positive bacteria exist widely in the human body, and they are harmful to the environment and human health. So, it is important to study the protein subcellular location of Gram-positive bacteria. There are a few researches on the protein subcellular location of Gram-positive bacteria. In 2007, Shen and Chou [8] established a Gram-positive bacteria dataset of five categories. They used the GO-PseAA discrete model and the Fusion OET-KNN method, and the overall success accuracy was 82.7% with the Jackknife test. In 2009, Shen and Chou [9] rebuilt the Gram-positive bacteria dataset with four categories: cell wall, cell membrane, cytoplasm, and extracell. The feature of gene ontology information and functional domain information were extracted, and the total success accuracy reached 82.2% with the Jackknife test. In 2012, the total success accuracy was 85.9% for the GP25 dataset constructed by Hu et al. [10]. In the 9th international conference on electrical and computer engineering, Rahman et al. [11] proposed two hybrid features, AACPPM and PAACPPM, which combined PPM with AAC and PseAAC, respectively. The accuracy of both AACPPM and PAACPPM were 73.2%. In 2017, Xiao et al. [12] took advantage of the dataset established by Shen and Chou in 2009 and applied the new algorithm, and a better result was obtained. In 2018, Xiao et al. [13] developed a new bias-reducing predictor. The results showed that this predictor was very helpful in predicting the training dataset.

In this paper, we reconstructed the Gram-positive bacterial protein subcellular location dataset. The amino acid composition information [14], the amino acid dipeptide composition information [15, 16], the gene ontology [17] annotation information, the hydropathy dipeptide [18] composition information, and the autocovariance average chemical shift [19] information were selected as characteristic parameters, then these parameters were combined. Finally, the overall accuracy in the Jackknife test was 86.1% by using the combined parameter AAC+DC+hpDC for the Support Vector Machine.

## 2. Materials and Methods

### 2.1. Dataset

In order to collect as much desired information as possible while ensuring a high quality for the dataset, the protein sequences were collected from the Swiss-Prot [20] database at http://www.uniprot.org/. The dataset was established in strict accordance with the following criteria: (1) We conducted a search for all protein sequences with “actinobacteria” and “firmicutes” in the OC firmicutes from the UniProtKB/Swiss-Prot database. (2) Different locations of the protein in the “Subcellular Location” annotation were selected, and the ambiguous or uncertain terms, such as “By similarity” and “Probably” were removed. (3) The protein sequence of 50 aa-3000 aa in the “Sequence” information were selected. (4) Sequences annotated by two or more locations were not included. (5) Sequences annotated with “fragment,” “B,” “X,” and “Z” were excluded. (6) To avoid any homology bias, the software CD-HIT [21] was used to winnow those sequences which have ≥25% sequence identity to any other sequence in the same subcellular location.

After completing the above steps, we obtained 700 Gram-positive proteins, and the specific distribution is shown in Table 1.

### 2.2. Amino Acid Composition (AAC)

The sequence information of proteins is the most basic feature information of all characteristic parameters [22]. The protein sequence consists of 20 amino acids (A, C, D, E, F, G, H, I, K, L, M, N, P, Q, R, S, T, V, W, and Y). The feature of the occurrence frequency of the 20 amino acids in the protein is important. So, the occurrence frequency of the 20 amino acids in the protein sequence can be selected as one of the characteristic parameters. The amino acid composition can be expressed as a 20-dimensional feature vector:

(1) $$\begin{array}{c} \text{AAC}={\left[f_{1},f_{2},f_{3},\cdots f_{20}\right]}^{T}, \end{array}$$

where *f*_*i*_ = (*n*_*i*_/*L*)(*i* = 1, 2, ⋯, 20), *n*_*i*_ is the occurrence number of the 20 native amino acids of the protein, *L* is the length of the protein, and *T* is the transpose operator.

### 2.3. Dipeptide Composition (DC)

One of the main drawbacks of the amino acid composition is that it only emphasizes on overall sequence information but ignores the sequence order information. In order to make full use of the sequence information of amino acids, we proposed using the amino acid dipeptide composition information. The amino acid dipeptide information is an improvement based on the AAC parameter, and it denotes the frequency of two adjacent amino acids in a 400-dimensional vector [23–25]. The dipeptide composition can be formulated as follows:

(2) $$\begin{array}{c} \text{DC}={\left[d_{1}\cdots d_{i}\cdots d_{400}\right]}^{T}, \end{array}$$

where *d*_*i*_(*i* = 1, 2 ⋯ 400) is the absolute occurrence frequencies of the 400 dipeptides and calculated by

(3) $$\begin{array}{c} d_{i}=\left(n_{i}/L-1\right), \end{array}$$

where *n*_*i*_ is the occurrence number of the 400 dipeptides of the protein and *L* is the length of the protein.

### 2.4. Gene Ontology (GO)

Gene ontology is a directed acyclic graph ontology widely used in bioinformatics, and gene ontology consists of three parts: biological process (P), molecular function (F), and cellular component (C). In the gene ontology database, we found that each AC number has a corresponding GO identification number: XXXXXXX. In this paper, since cellular component (C) contains the location information of a protein, in order to ensure the accuracy of the prediction, only biological process (P) and molecular function (F) were extracted.

The specific steps are as follows:

**Step 1 .**

The “Text” documents of all protein sequences were downloaded in Swiss-Port, and the annotation information of all biological processes (P) and molecular functions (F) was extracted.

**Step 2 .**

BLAST [26] was used to find homologous sequences of biological process (P) and molecular function (F) without annotation information. The homology threshold was set to 60%, and the *E* value was set to 0.001.

**Step 3 .**

The frequency of occurrence of each GO term was calculated:

(4) $$\begin{array}{c} f=\frac{N_{x}^{i}}{N_{x}}, \end{array}$$

where *N*_*x*_^*i*^ denotes the frequency of the *i*th GO terms at the *x* position of Gram-positive bacteria and *N*_*x*_ is the total number of amino acid sequences at the *x* position of Gram-positive bacteria. A threshold value*T*was set; when *f* > *T*, the corresponding GO terms were retained.

**Step 4 .**

The GO terms of all target sequences were integrated and repeated, then 2573 GO terms were acquired. Finally, the 2573 GO terms were integrated into one vector, *P*_GO_:

(5) $$\begin{array}{c} P_{\text{GO}}=\left\{\psi_{1},\psi_{2},\cdots \psi_{n},\cdots \psi_{2573}\right\}, \end{array}$$

where *ψ*_*n*_ is 0 or 1, and the GO number with the corresponding location information of the proteins was set to 1; otherwise, it was 0.

### 2.5. Autocovariance Average Chemical Shifts (acACS)

The most important issue is how to extract features from primary sequences of a protein in a predictor. Hence, the acACS [27, 28] algorithm that uses simple secondary structure information to represent the sample of a protein was proposed. The average chemical shift of a protein is closely related to the protein's secondary structure [29] and the function of this protein. The secondary structure of the protein sequence (C, H, and E) was obtained by submitting the protein sequence to the PSIPRED (http://bioinf.cs.ucl.ac.uk/psipred/) online tool, and then the secondary structure was submitted to Fan et al.'s [30] average chemical shift service website acACS (http://wlxy.imu.edu.cn/college/biostation/fuwu/acACS/index.asp) to obtain the results of the chemical shifts. For a protein *P*

(6) $$\begin{array}{c} P=\left[j_{1},j_{2}\cdots j_{i}\cdots j_{L}\right], \end{array}$$

where *L* means the length of the protein sequence *P* and *j* is the 20 amino acid residues; thus, *P* can be expressed as follows:

(7) PacACSi=ψi0,ψi1,ψi2,⋯,ψiλ, i=N15,C13α,H1α,H1N, 0<λ<L,

where *ψ*^*i*^(*λ*) represents the correlation factor of the average chemical shift for *j*_*l*_ with the average chemical shift for *j*_*l*+*λ*_ along the protein sequence. The factor *λ*(0 < *λ* < *L*) means the rank of correlation. The factor *i* can be represented in a different composition of ^15^N, ^13^C_*α*_, ^1^H_*α*_, and ^1^H_*N*_. In order to obtain the best accuracy, an appropriate number factor *λ* and the best combination mode *i* were selected to predict the results.

### 2.6. Hydropathy Dipeptide Composition (hpDC)

Hydropathy dipeptide composition is based on the improvement of hydrophilic and hydrophobic proteins. Firstly, 20 kinds of amino acids were divided into 6 categories [31] according to the hydrophilic and hydrophobic standards, namely, strong hydrophilic amino acids (H), strong hydrophobic amino acids (L), weak hydrophilic amino acids or weak hydrophobic amino acids (W), and three types of proline (P), glycine (G), and cysteine (C) with special chemical structures. Hydrophilic and hydrophobic dipeptide composition is a discrete method that uses protein sequence representation, and it can be represented as a 36-dimensional vector:

(8) $$\begin{array}{c} P_{\text{hpDC}}={\left[q_{1}\cdots q_{i}\cdots q_{36}\right]}^{T}, \end{array}$$

where *q*_*i*_ = (*n*_*i*_/*L* − 1) (*i* = 1, 2 ⋯ 36) represents the occurrence frequencies of the 36 hydropathy dipeptides, while *n*_*i*_ denotes the occurrence number of the 36 hydropathy dipeptides of the protein and *L* is the length of the protein.

### 2.7. Support Vector Machine (SVM)

The Support Vector Machine is a machine learning method to solve classification and regression problems based on statistical principles. The SVM model is a representation of the examples as points in space, mapped by a kernel function so that the examples are divided by a clear gap that is wide enough. The new examples are mapped into the same space and predicted according to which side of the gap they fall on. The radial basis kernel function (RBF) was used to obtain the best classification hyperplane. The regularization parameter *C* and the kernel width parameter *γ* were tuned via the grid search method. So far, the risk minimization of the SVM algorithm has become the latest research hotspot and it has been successfully applied to various fields [32–38], especially in the field of biological computing, such as in the prediction of protein sequence structure and in the classification of protein structure [28, 39–46]. In this paper, the LIBSVM algorithm has been used to predict various feature information, which can be downloaded from http://www.csie.ntu.edu.tw/cjlin/libsvm/.

## 3. Results

### 3.1. Cross-Validation

In statistical prediction, three test methods of prediction accuracy are used: the Jackknife test, the*k*-fold cross-validation test, and the independent test [8, 47–53]. In this paper, a strict and objective method for the Jackknife test was adopted to examine the performance of the proposed model. The principle of the Jackknife test is to select one from among all protein sequences as a testing set and the other remaining sequences as a training set until all protein sequences are recycled once.

### 3.2. Evaluation of the Predictive Performances

In order to evaluate the performance of related predictive methods and the reliability of the algorithm, the sensitivity (*S*_*n*_), specificity (*S*_*p*_), accuracy (ACC), Matthew's correlation coefficient (MCC), and overall accuracy (OA) [54–59] were used and defined by

(9) $$\begin{array}{c} S_{n}=\frac{\text{TP}}{\left(\text{TP}+\text{FN}\right)},\\ S_{p}=\frac{\text{TN}}{\left(\text{TN}+\text{FP}\right)},\\ \text{ACC}=\frac{\left(\text{TP}+\text{TN}\right)}{\left(\text{TP}+\text{TN}+\text{FP}+\text{FN}\right)},\\ \text{MCC}=\frac{\left(\text{TP}\times \text{TN}\right)-\left(\text{FP}\times \text{FN}\right)}{\left(\text{TP}+\text{FN}\right)\times \left(\text{TN}+\text{FN}\right)\times \left(\text{TP}+\text{FP}\right)\times \left(\text{TN}+\text{FP}\right)},\\ \text{OA}=\overset{4}{\underset{i=1}{\sum }}\frac{{\text{TP}}_{i}}{N}, \end{array}$$

where *N* is the total number of protein sequences in the dataset, TP represents the numbers of the correctly recognized positives, FN is the numbers of the positives recognized as negatives, FP means the numbers of the negatives recognized as positives, while TN is the numbers of correctly recognized negatives.

### 3.3. The Prediction of Gram-Positive Bacteria

In this paper, in order to investigate the effectiveness of our approaches, we have used five feature extraction strategies and the SVM is used as classification algorithm.

**Figure 1:**
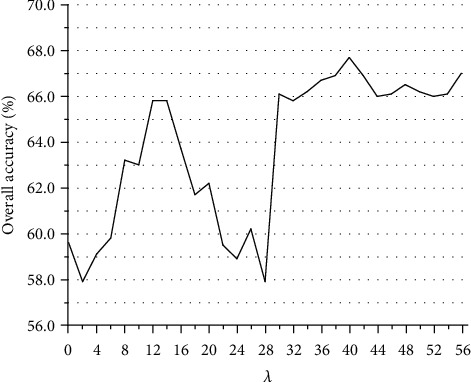
Predictive results with respect to the correlation factor *λ* of the acACS based on the Jackknife test. The best results obtained with *λ* = 40.

**Figure 2:**
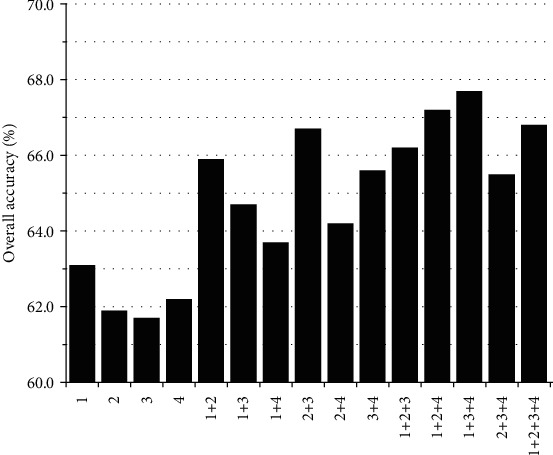
The combination scheme of chemical shifts. The number 1 denotes ^1^H_*α*_, 2 denotes ^1^H_*N*_, 3 denotes ^15^N, and 4 denotes^13^C_*α*_.

The autocovariance average chemical shift (acACS) vectors were formed based on protein sequence, and in order to obtain the best prediction results, we need to find the best chemically shifted atom combination and the best parameter *λ*.  Figure 1 shows that the predicted results for *λ* ranges from 0 to 56, and the best *λ* is 40.  Figure 2 shows that the prediction result was the best when the combination mode of chemically shifted atoms was ^1^H_*α*_ + ^15^N + ^13^C_*α*_. For gene ontology information, the first 2573-dimensional vector was obtained. Since the redundancy of data has a detrimental effect on the prediction results, we used the method of principal component analysis to reduce the vector to 854 dimensions. First of all, the 2573 GO terms were integrated into one vector, then the frequency of each GO term was counted. According to the sum of frequencies, the first 854 data was selected.

The predicted results by the Jackknife test for the different information parameters are recorded in Table 2, and the predicted results based on the combined parameter information with the Jackknife test are shown in Table 3. The results showed that the combined parameters were better than a single characteristic parameter. And the combined parameter AAC+DC+hpDC obtained the best accuracy which was 86.1%. The results indicated that the combined parameter was helpful to predict the protein subcellular location of Gram-positive bacteria. The reason that the accuracies of AAC+GO+acACS+hpDC, AAC+DC+GO+hpDC, and AAC+DC+GO+acACS+hpDC were lower than AAC+DC+hpDC was probably due to the redundancy of data.

## 4. Discussion

For the purpose of comparing the predictive capability of our method, the predicted results of Shen's, Hu's, and Julia Rahman's method are enumerated in Table 4. It can be seen from Table 4 that our results were superior to others. The accuracy of our method was 3.4% higher than Shen's first work, 3.9% higher than Shen's second work, 0.2% higher than Hu's work, and 12.9% higher than Julia Rahman's work.

Gram-positive bacteria exist widely in nature and could cause many diseases, so studying Gram-positive bacteria subcellular location could solve the many problems of disease. In this paper, the dataset of protein subcellular location of Gram-positive bacteria was reconstructed, and the subcellular location of Gram-positive bacterial protein was predicted. The method in this paper had the advantages of a simple algorithm and an automatic process. The results showed that the combined parameter can improve the prediction accuracy of protein subcellular location of Gram-positive bacteria.

The protein data used to support the findings of this study are included within the supplementary information file.

## Figures and Tables

**Table 1:** Dataset of Gram-positive bacteria subcellular location proteins.

Subcellular location	Number of proteins
Cell wall	22
Extracell	214
Cytoplasm	252
Cell membrane	212
Total	700

**Table 2:** The predictive results based on the different information parameters in the Jackknife test.

Features		Location				OA (%)
		Cell wall	Extracell	Cytoplasm	Cell membrane	
AAC	*S*_*n*_ (%)	13.64	74.30	70.64	85.85	74.6%
	*S*_*p*_ (%)	99.85	84.77	88.62	89.34	
	MCC	0.31	0.58	0.61	0.73	
	ACC (%)	96.71	81.57	82.14	88.29	
DC	*S*_*n*_ (%)	0.00	70.09	70.24	84.91	72.4%
	*S*_*p*_ (%)	99.85	84.57	86.34	88.53	
	MCC	-0.01	0.54	0.57	0.71	
	ACC (%)	96.71	80.14	80.57	88.53	
GO	*S*_*n*_ (%)	0.00	75.23	71.03	66.98	68.9%
	*S*_*p*_ (%)	99.71	86.63	82.14	85.45	
	MCC	-0.01	0.61	0.53	0.52	
	ACC (%)	96.57	83.14	78.14	79.86	
acACS	*S*_*n*_ (%)	0.00	66.36	70.24	73.11	67.7%
	*S*_*p*_ (%)	99.95	83.13	80.36	88.53	
	MCC	-0.01	0.49	0.50	0.62	
	ACC (%)	96.85	78.00	76.71	83.86	
hpDC	*S*_*n*_ (%)	0.00	73.82	76.59	76.42	73.3%
	*S*_*p*_ (%)	99.85	86.01	82.81	91.60	
	MCC	0.07	0.59	0.59	0.69	
	ACC (%)	96.71	82.3	80.57	87.00	

**Table 3:** The predictive results based on the hybrid information in the Jackknife test.

Features		Location				OA (%)
		Cell wall	Extracell	Cytoplasm	Cell membrane	
AAC+GO	*S*_*n*_ (%)	9.09	87.85	76.59	86.79	81.0%
	*S*_*p*_ (%)	99.56	89.10	92.86	90.78	
	MCC	0.18	0.75	0.71	0.76	
	ACC (%)	96.71	88.71	87.00	89.57	
AAC+hpDC	*S*_*n*_ (%)	40.91	83.18	83.33	89.60	83.9%
	*S*_*p*_ (%)	99.26	90.74	91.96	94.50	
	MCC	0.50	0.74	0.78	0.74	
	ACC (%)	97.14	87.26	88.94	89.24	
AAC+GO+acACS	*S*_*n*_ (%)	22.73	85.51	81.35	86.79	82.4%
	*S*_*p*_ (%)	99.56	90.74	91.74	92.21	
	MCC	0.37	0.75	0.74	0.78	
	ACC (%)	97.14	89.14	88.00	90.57	
AAC+DC+hpDC	*S*_*n*_ (%)	40.91	86.92	87.30	88.68	86.1%
	*S*_*p*_ (%)	99.71	91.15	92.86	93.65	
	MCC	0.49	0.77	0.77	0.82	
	ACC (%)	97.57	89.96	89.57	92.14	
AAC+GO+acACS+hpDC	*S*_*n*_ (%)	22.73	83.65	86.51	85.85	83.4%
	*S*_*p*_ (%)	99.56	90.71	90.63	94.67	
	MCC	0.37	0.73	0.77	0.81	
	ACC (%)	97.14	88.57	89.14	92.00	
AAC+DC+GO+hpDC	*S*_*n*_ (%)	36.36	83.18	82.54	91.98	84.1%
	*S*_*p*_ (%)	99.41	88.86	92.86	93.24	
	MCC	0.48	0.74	0.76	0.84	
	ACC (%)	97.43	88.86	89.14	92.86	
AAC+DC+GO+acACS+hpDC	*S*_*n*_ (%)	22.27	84.11	84.13	90.09	84.1%
	*S*_*p*_ (%)	99.71	90.95	92.86	93.24	
	MCC	0.44	0.74	0.78	0.82	
	ACC (%)	97.43	88.86	89.71	92.29	

**Table 4:** The results compared with previous methods.

Method	Validation method	OA (%)
Shen's first work^a^	Jackknife test	82.7%
Shen's second work^b^	Jackknife test	82.2%
Hu's work^c^	Jackknife test	85.9%
Julia Rahman's work^d^	8-Fold cross-validation	73.2%
This study	Jackknife test	86.1%

^a^See ref. [8]. ^b^See ref. [9]. ^c^See ref. [10]. ^d^See ref. [11].

## Data Availability

The data used to support the findings of this study are available from the corresponding author upon request.

## Supplementary Materials

Supplementary Materials The protein data used to support the findings of this study are included within the supplementary information file.
