# Supplementary material for: Predicting Gram-Positive Bacterial Protein Subcellular Location by Using Combined Features
Source: Biomed Res Int. 2020 Aug 2;2020:9701734. doi: 10.1155/2020/9701734 (PMC7421015; doi:10.1155/2020/9701734)
Supplement: Supplementary Materials — The protein data used to support the findings of this study are included within the supplementary information file. [file 9701734.f1.pdf]

The protein data used to support the findings of this study are included within the supplementary information file.

Dataset of Gram-positive bacteria subcellular location proteins

1、Cell wall(22)

>P9WIF5 >Q79FW5 >P9WIU5 >P9WMK7 >A1A278 >P42829 >P9WP28 >A0QPU4 >A9WNA0 >L7N675 >Q79FL8 >Q6MWV0 >P55111 >Q79FS8 >P0CZ00 >Q79G04 >P9WLN3 >O34313 >O07532 >Q8KQR1 >Q9L7Q2 >Q07833

2、 Extracell (214)

>Q8KRU5 >O53289 >O53361 >P9WIB9 >P9WQB1 >O05442 >P9WPE7 >P9WFK7 >P80561 >K7N5M8 >P9WJE1 >P9WLL7 >Q93MW7 >Q3L8N0 >G2NFJ9 >P9WJA3 >P9WIR7 >E5KIB6 >O53168 >P9WJD9 >Q82P90 >P0C8P9 >P9WKY5 >P9WII3 >Q47KB1 >I6Y2J4 >P94248 >P9WJD7 >P9WJD5 >P9WJC3 >Q79FE1 >Q6M6N7 >I6XEI5 >I6X8R5 >P96917 >P96463 >Q54410 >P83615 >P86242 >P9WIG7 >P9WNI7 >P83543 >P9WIR3 >O53692 >P9WF83 >P9WF93 >A0QNJ6 >P53647 >P86325 >O69622 >A0QNJ5 >L7N695 >Q79FB3 >I6X486 >O69623 >P71623 >P83544 >P9WF59 >P22266 >O50393 >Q45296 >O53501 >A4QD57 >A4QFQ3 >P9WP38 >P9WF53 >P30343 >P9WFA1 >I6YC53 >P9WJ33 >Q8NQ03 >P0C216 >P10477 >Q9X721 >P40136 >P42293 >P94522 >O34344 >O31422 >P54422 >O33599 >Q183R7 >A6QIG7 >P15917 >P0C2S5 >P68577 >P0C0J0 >P39844 >Q8GJ44 >P21543 >P0C1U8 >P45796 >P10547 >O31527 >O87236 >P39116 >O87237 >P05656 >P86475 >Q2FZL3 >P86476 >Q8GCB2 >P54507 >Q08002 >P25152 >Q04707 >P06279 >P83513 >H2A7G5 >P45741 >P26831 >P37957 >Q60136 >P0C2S1 >O85465 >Q45070 >O33635 >P04957 >P00691 >Q06851 >P17692 >E9K9Z1 >Q2FXC3 >D3JTC1 >Q2QBT0 >O82833 >Q60053 >P94449 >P80696 >A0P8X0 >Q9KWM4 >Q5YLG1 >B2MVM5 >Q931F4 >P34071 >A9Q0M7 >P80057 >P23564 >P86720 >P10335 >P29148 >P16699 >P0A0M0 >P38422 >Q838U8 >Q8Y9T5 >P0C1U7 >P00649 >P06653 >P28842 >P29141 >Q93M42 >P10424 >Q0Z8B6 >P80172 >P29767 >P11001 >P14892 >P10481 >P39800 >P37710 >P37134 >Q9RLV9 >P14262 >P43131 >Q46134 >Q4MV79 >P33673 >P40943 >P50864 >Q6RUF5 >A5H1G9 >O31982 >P39738 >P43270 >P23382 >P11701 >Q53591 >P0CY50 >O07921 >P24556 >P68569 >P36550 >Q45882 >Q8GPI4 >Q9ZNI1 >Q05622 >P16397 >P68802 >P38578 >O66037 >P42983 >P06886 >Q2FXU3 >Q9KJT6 >Q2FWV6 >P16396 >Q5HEA4 >P0C047 >P45704 >P96600 >Q02551 >P16169 >P35518 >P09879 >Q2G222 >Q03091 >Q4L980 >A6QG59 >A6QG57 >Q2FWP0 >Q9K6A3 >Q06242 >P94576

3、 Cytoplasm (252)

>P9WIL5 >P9WIL7 >Q9AGJ6 >P9WQP7 >P9WMJ9 >P9WP19 >P9WIC1 >P9WPP9 >P9WPY3 >P9WKH1 >P9WGK3 >P9WP49 >P18326 >Q9L9D7 >Q9ZN78 >P9WN39 >P9WGI9 >Q9S426 >P9WPH9 >L0T905 >P71590 >P83221 >P9WHQ9 >Q9L9F1 >P74838 >P53627 >O07776 >O51917 >A0QVQ8 >P9WMJ5 >A0R006 >P9WPZ5 >Q8GED9 >A0QZ12 >Q9WX14 >Q54206 >D2PPM7 >P9WMN9 >A0QNJ2 >P50979 >P9WIL1 >P0DKS9 >P07128 >Q8CJR5 >Q9X7R6 >Q8NNX7 >P24221 >P9WP99 >P9WH01 >P46712 >O53193 >P0DKS5 >P27785 >O69473 >Q8G4X4 >O66126 >P9WPH7 >O68887 >Q9K492 >Q9RJN1

>P9WMT3 >P9WQ55 >A0QTV4 >Q8G4U8 >P9WIA3 >P48859 >Q4JVU0 >Q8NP66 >P94965 >A5TZU0
>O31678 >O31749 >P71447 >G3KIM8 >P37573 >P35149 >O31616 >Q9S4K9 >O31775 >P39644
>P31005 >P32397 >P27623 >P53555 >O34559 >Q6G7I0 >P54322 >P42305 >P16304 >P50849
>P37570 >Q97ML3 >P08877 >Q8KY51 >P19080 >P11959 >Q9ZJ19 >P13799 >P26380 >P37552
>P94527 >P94388 >P40859 >P0DKY4 >P13800 >P39779 >Q2FVN3 >P42085 >P11570 >Q92EU6
>P11569 >P11931 >P12010 >P26379 >Q8CWP9 >P56968 >Q9L4G1 >P19669 >O32271 >P33166
>Q9FBG4 >P41972 >O31743 >P13484 >P97030 >P71011 >Q9CGF7 >H6LGM8 >P45494 >Q7WZY5
>Q9KEI9 >P36946 >P39153 >O31423 >Q59112 >Q8RJB2 >Q9K8V3 >P39795 >Q92EU4 >P0A0C2
>P46107 >O34484 >Q9R9H8 >H6LGM7 >P39813 >P96692 >Q797E6 >Q5HF86 >P32396 >P22326
>O31784 >Q92EU3 >P54272 >P0A4K2 >Q7A338 >O31562 >Q9S6S1 >A9KM56 >P06555 >Q9EYW6
>O32102 >P39638 >O05250 >O68575 >Q92ET9 >O34767 >Q47840 >Q9Z4P6 >Q8YAS7 >P46908
>O34543 >P36772 >Q97II1 >P0CC08 >P23660 >O05516 >Q67QF2 >O07874 >Q5HJF4 >P50846
>Q92EU2 >P37584 >P21885 >P40830 >P13375 >O83018 >Q8KWT5 >P37563 >P40408 >P39802
>P23445 >Q02169 >P17889 >Q9L4Q8 >O05505 >P15874 >P96677 >O32215 >Q60023 >Q7A2M4
>Q9KCQ4 >Q47839 >Q0AVA8 >Q10744 >P03000 >P94548 >P45359 >P94433 >O34703 >P96711
>P30053 >P29441 >P68815 >P54262 >O34825 >P80870 >P58253 >O34769 >P30363 >O34685
>Q2G0D1 >Q5XDW4 >Q0AVM5 >O05506 >Q9CFY8 >Q45056 >O52951 >Q8GND0 >Q9ZB09 >Q5HJF7
>P13522 >O31517 >P40759 >Q2G1T7 >Q02147 >P16400 >P60611 >P54495 >P51591 >P0A0I7
>O05517 >Q9X315 >Q9CH00 >Q46171 >Q99171 >P27547 >O32266 >P54264 >O31601 >Q48768
>P39486 >Q7A2R2

4、Cell membrane（212）

>P0A303 >P9WHS7 >P9WL75 >Q8RQL4 >P9WQM7 >P06109 >P9WG12 >L0T550 >P68434 >P65713
>P9WP92 >P9WFZ7 >P9WGN5 >P9WP47 >P9WQ28 >P9WLI1 >P9WM87 >P9WG15 >P59950 >P9WQ44
>P9WQ52 >P9WQJ3 >P63350 >Q7TZ67 >P9WFP1 >Q7D745 >P9WJI3 >P9WMX0 >P0A627 >P9WQI6
>P9WG62 >P9WMX4 >P9WJX2 >P9WJ98 >P9WQ65 >P9WFZ9 >P9WM19 >P9WJY4 >Q6NJ43 >P53425
>P9WG90 >P9WLS5 >P9WLC7 >P9WG89 >P9WJW7 >P9WFP3 >P9WL79 >P9WFM5 >P9WM35 >P9WJX8
>P9WN98 >P9WG87 >P9WJX4 >P9WGF5 >P9WFM3 >P9WGF6 >P40180 >O06079 >P9WJB0 >P9WJB2
>P9WJY0 >P46838 >P9WK33 >P9WIW3 >P9WG84 >Q9ADP8 >P9WLY1 >P9WFN7 >P9WJX7 >P42531
>P9WJX1 >P9WLI5 >P9WJT6 >P9WLW1 >P53426 >P65371 >P9WJD2 >P9WLL5 >O88022 >P54880
>P9WM06 >P9WGU4 >P66884 >O33057 >P9WLG3 >B7GPC7 >P9WHW5 >P9WGT7 >O53493 >P9WI83
>A0QWG6 >P9WI79 >P9WI67 >P9WI81 >O65934 >P9WJ67 >P9WGE9 >O69729 >P9WG29 >P9WNZ3
>P9WFF7 >P9WQ35 >P9WNF9 >P9WP15 >P9WQ37 >P9WJB5 >P9WQJ9 >O53585 >P9WQJ7 >I6Y9J2
>P9WGK9 >P9WQN3 >D1A4G7 >P9WN23 >P9WJK3 >Q5YXD6 >P9WPU1 >P9WQL9 >L0T911 >P9WK47
>P9WJQ1 >O07777 >P9WKD1 >P9WJY7 >P9WJ71 >P9WGK7 >Q8NLB7 >P9WP37 >A0R211 >Q8NQC8
>P9WN97 >Q740J9 >P9WP71 >P9WPF7 >P9WGJ3 >P9WGL3 >A0R7G2 >P9WQI9 >P9WN05 >B7GPC7
>P9WH23 >P9WQI3 >P9WIZ5 >P9WIQ7 >P9WG23 >P9WN01 >P9WG21 >A0QIR4 >P9WMB5 >Q8NN75

>P9WPG3 >Q5YWD4 >A0QNF5 >A5U7B6 >Q742C0 >Q8NTW4 >A0QWG5 >A0QNZ7 >P9WJF3 >Q46072
>P9WPS5 >A0QWU7 >A0R036 >Q8FNR0 >P25048 >P9WPS7 >Q8G847 >Q79VC4 >O69690 >P9WHR1
>P9WPG5 >P9WGU3 >P32011 >O33192 >A0QR01 >P9WP67 >P0A4I7 >Q9ZEP3 >O53857 >P9WPN7
>P9WPS8 >Q5YZ37 >P9WPT7 >P96454 >O53945 >Q8G846 >P9WGU0 >P9WIW4 >P9WPT1 >P9WNR5
>Q8NS49 >H8F2P5 >Q9CC08 >P9WG11 >P9WMX1 >Q9XAR5 >P9WPG1 >P71485 >P31502 >P9WPL7
>P22409 >Q9F7Y9 >K4REQ6 >Q9KYS0 >O06342 >Q50098 >Q00718 >A1ULE2 >O24723 >P9WNQ5
>P48242 >P65822
